# Significant radiative impact of volcanic aerosol in the lowermost stratosphere

**DOI:** 10.1038/ncomms8692

**Published:** 2015-07-09

**Authors:** Sandra M. Andersson, Bengt G. Martinsson, Jean-Paul Vernier, Johan Friberg, Carl A. M. Brenninkmeijer, Markus Hermann, Peter F. J. van Velthoven, Andreas Zahn

**Affiliations:** 1 Department of Physics, Lund University, P.O. Box 118, Lund 22100, Sweden; 2 Science Systems and Applications, Inc., Parkway, Suite 200, Hampton, Virginia 23666, USA; 3 NASA Langley Research Center, 11 Langley Boulevard, Hampton, Virginia 23681, USA; 4 Max Planck Institute for Chemistry, Department of Atmospheric Chemistry, Hahn-Meitner Weg 1, Mainz 55128, Germany; 5 Leibniz Institute for Tropospheric Research, Department of Experimental Aerosol and Cloud Microphysics, Permoserstrasse 15, Leipzig 04318, Germany; 6 Royal Netherlands Meteorological Division of Chemistry and Climate, Climate Research, P.O. Box 201, De Bilt 3730AE, The Netherlands; 7 Institute for Meteorology and Climate Research, Atmospheric Trace Gases and Remote Sensing, Karlsruhe Institute of Technology (KIT), P.O. Box 3640, Karlsruhe 76021, Germany

## Abstract

Despite their potential to slow global warming, until recently, the radiative forcing associated with volcanic aerosols in the lowermost stratosphere (LMS) had not been considered. Here we study volcanic aerosol changes in the stratosphere using lidar measurements from the NASA CALIPSO satellite and aircraft measurements from the IAGOS-CARIBIC observatory. Between 2008 and 2012 volcanism frequently affected the Northern Hemisphere stratosphere aerosol loadings, whereas the Southern Hemisphere generally had loadings close to background conditions. We show that half of the global stratospheric aerosol optical depth following the Kasatochi, Sarychev and Nabro eruptions is attributable to LMS aerosol. On average, 30% of the global stratospheric aerosol optical depth originated in the LMS during the period 2008–2011. On the basis of the two independent, high-resolution measurement methods, we show that the LMS makes an important contribution to the overall volcanic forcing.

## Abstract (web summary)

The role played by volcanic-induced cooling in the recent warming hiatus is not accurately described in the latest phase of the Coupled Model Intercomparison Project. Here, the authors use satellite and aircraft data to investigate the radiative impact of volcanic aerosols in the lowermost stratosphere since the year 2000.

## Graphical abstract

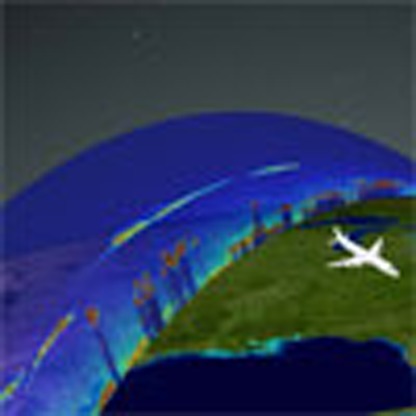

Early 21th century global warming has been overestimated by almost all simulations of historical climate change in the latest phase of the Coupled Model Intercomparison Project (CMIP5)^1^,^2^. This divergence between simulated and observed warming rates could be evidence of serious model errors in the climate sensitivity to anthropogenic greenhouse gas increases^3^, or to systematic model deficiencies in representing natural internal variability^4^,^5^. It has been shown that tropical Pacific cooling due to increased subduction and upwelling^6^,^7^ and variations in solar^2^ and volcanic aerosol forcing^5^,^8^ contribute to the discrepancy because they are not realistically described in CMIP5 simulations of recent climate change. Volcanic eruptions induce ‘persistent variability' in the stratospheric aerosol layer^8^,^9^,^10^. Sulfur dioxide from volcanic eruptions forms sulfate particles that reflects sunlight back to space, exerting a cooling effect^11^. The CMIP5 historical simulations did not account for observed increases in volcanic aerosol loadings after the year 2000.

Most satellite-based estimates of global stratospheric aerosol optical depth (AOD) rely on occultation and other limb viewing measurements made at altitudes above the 380 K potential temperature level (on average, above 15-km altitude)^9^,^10^,^12^. These data have been used in a wide range of different climate studies, but do not include the lowermost stratosphere (LMS)^5^,^8^,^13^,^14^,^15^. The LMS lies between the 380 K potential temperature level (at ∼17-km altitude in the tropics and 14 km at mid-latitudes) and the underlying tropopause (coinciding with the 380 K potential temperature level in the tropics and at ∼10 (11) km at mid-latitudes in winter (summer)), and constitutes over 40% of the stratospheric mass^16^. The stratosphere above 380 K potential temperature is connected to the troposphere by an upward flow across the tropical tropopause via the Brewer–Dobson circulation. At mid- and high latitudes, the LMS receives seasonally varying fractions of subsiding stratospheric air from higher altitudes, and is also affected by tropospheric air crossing the extratropical tropopause. These flow patterns cause characteristic concentration gradients of trace gases^17^,^18^ and aerosols^19^. Volcanic aerosols reach the LMS either by direct injection in the extratropics or by transport from above via the Brewer–Dobson circulation^18^.

Global estimates of the contribution from volcanism to stratospheric aerosols have historically been performed by limb viewing satellite instruments, such as SAGE II^20^ (Stratospheric Aerosol and Gas Experiment), GOMOS^21^ (Global Ozone Monitoring by Occultation of Stars) and presently OSIRIS^22^ (Optical Spectrograph and Infrared Imaging System). Their long line of sight is obscured by the occurrence of clouds close to the tropopause and by dense volcanic clouds. These problems make it difficult to use OSIRIS for observations below 380 K potential temperature^23^,^24^, and also limit the use of SAGE II^25^.

Recently, it has been suggested that the LMS contributes significantly to stratospheric AOD^24^. For the time period from 2000 to 2013, Ridley *et al.*^26^ estimated that 30–70% of the total stratospheric AOD was from volcanic aerosols in the LMS. The latter study made use of ground-based lidar retrievals, the Aerosol Robotic Network (AERONET) of sun photometers, and balloon-borne measurements. The lack of vertically resolved aerosol information in the AERONET measurements limits their ability to reliably partition the tropospheric and stratospheric contributions to the AOD.

The results presented here rely on independent observational measurements from two different sources. The first source is the nadir viewing lidar on the Cloud-Aerosol Lidar and Infrared Pathfinder Satellite Observation (CALIPSO)^27^ satellite. This instrument measures from the stratosphere down to the ground with high vertical resolution, thus enabling aerosol observations in the upper troposphere (UT) and LMS. The second source of information is from analyses of aerosol samples collected by the IAGOS-CARIBIC program (In-service Aircraft for a Global Observing System—Civil Aircraft for the Regular Investigation of the atmosphere Based on an Instrument Container), a passenger aircraft-based observatory^28^. IAGOS-CARIBIC was operational for most of the period 1999–2013, permitting study of the LMS in the Northern Hemisphere (NH). The global stratosphere up to 35-km altitude was investigated with CALIPSO during the period 2008 to early 2012, which covers several of the larger volcanic eruptions. We use the 2008 Kasatochi eruption to study the detailed post-eruption vertical structure of the LMS aerosol. Our study attempts to quantify the radiative influence of the LMS aerosol relative to the radiative impact of total stratospheric AOD (measured at a wavelength of 532 nm). In contrast to previous work, we explicitly resolve the vertical ‘fine structure' of the aerosol loadings after a series of volcanic eruptions. During the period 2008–2011, volcanic activity was most pronounced in the NH. The Southern Hemisphere (SH) was generally close to background conditions during this period, showing radiative properties that can be explained by stratospheric circulation and stratosphere–troposphere exchange.

## Results

### IAGOS-CARIBIC observations

**Figure 1:**
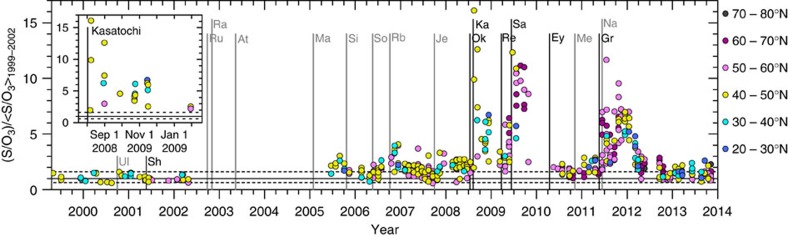
Identification of volcanic aerosol in the LMS. IAGOS-CARIBIC time series of S/O_3_ (ng m^−3^ STP p.p.b. v^−1^) in the LMS, normalized by average S/O_3_ during the 1999–2002 period of low volcanic influence. Major tick marks relate to Jan 1. The measurements were made at 9–12-km altitude, and the marker colour indicates the latitude band of aerosol sampling. Each measurement point corresponds to 100 (150 in 1999–2002) min of aerosol sampling. The full line indicates the geometrical average and the dashed lines the minimum and maximum S/O_3_ ratio during the 1999–2002 period, normalized to its geometrical average of that period. The start dates of tropical (grey) and NH extratropical (black) eruptions that affected the stratosphere of the NH are denoted by vertical lines. The eruptions are: Ul (Ulawun), Sh (Sheveluch), Ru (Ruang), Ra (Reventador), At (Anatahan), Ma (Manam), Si (Sierra Negra), So (Soufrière Hills), Rb (Rabaul), Je (Jebel at Tair), Ok (Okmok), Ka (Kasatochi), Re (Redoubt), Sa (Sarychev), Ey (Eyjafjallajökull), Me (Merapi), Gr (Grimsvötn) and Na (Nabro), see Table 1 for details. The inset gives details for the Kasatochi eruption.

IAGOS-CARIBIC observations are performed regularly at altitudes of 9–12 km, which is in the free troposphere in the tropics and in the UT/LMS in the extratropics. Measurements during intercontinental flights provided sampling of the LMS in the NH. The majority of these samples (90%) were collected between 30° N and 65° N. To identify volcanic influence on aerosol sampled in the LMS, we use S/O_3_, the ratio of particulate elemental sulfur to *in situ* ozone. This is a powerful tracer^29^ because the (non-volcanic) background S/O_3_ ratio is set high in the stratosphere before transport into the LMS. The S/O_3_ time series in the LMS (Fig. 1), obtained from near-monthly intercontinental IAGOS-CARIBIC flights, show clear evidence of influence from volcanic eruptions (Table 1). After a period of little volcanic influence during 1999–2002, large S/O_3_ ratios (increases of up to a factor of 16 relative to background) were measured following three extratropical eruptions between 2008 and 2011. Elevated S/O_3_ is also associated with a number of tropical eruptions between 2005 and 2012 (ref. 30). The effects of tropical eruptions on the mid- to high-latitude LMS region appear a few months to more than a year after the eruptions, due to the time required for transport from the tropics^18^. In 2013, the LMS aerosol concentrations again approached the background levels of 1999–2002.

Volcanic aerosol from the eruption of Kasatochi in August 2008 (see insert in Fig. 1) was first measured by the CARIBIC observatory over eastern Europe 1 week after the eruption^29^. Samples collected within 2 months after the eruption showed large variation in their S/O_3_ ratios, while those collected after longer than 2 months were more homogeneous as a result of mixing in the atmosphere. Elevated S/O_3_ ratios indicate that the LMS was influenced by Kasatochi at least until March 2009, seven months after the eruption. The S/O_3_ ratios increased again following multiple eruptions of the Redoubt volcano in March/April 2009.

### CALIPSO observations

**Figure 2:**
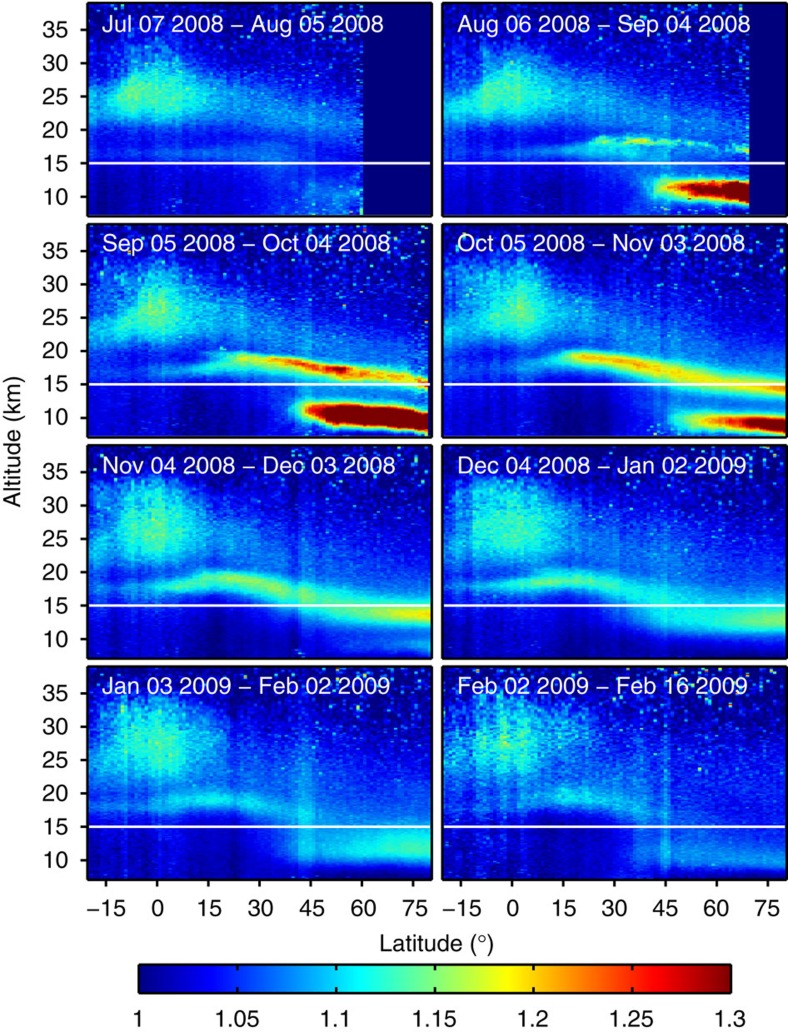
Latitude and altitude distributions of Kasatochi volcanic aerosol. Distribution of the Kasatochi volcanic aerosol from CALIPSO measurements. Results are monthly and zonally averaged scattering ratios ((measured total scattering)/(modelled air molecular scattering)) from July 2008 to Feb 2009. Positive latitude values refer to the NH and negative to the SH. For Feb 2009, only 2 weeks of data were available. The feature in the tropics at 25 km, which is enhanced in the scattering ratio due to the weak scattering from air molecules at high altitudes, is already present before the eruption of Kasatochi, and is likely related to tropical upwelling and particle formation. High-latitude data are missing in the top two panels because of the limited latitudinal extent of the CALIPSO night-time data during the summer season. The white line indicates 15-km altitude.

While the majority of CARIBIC measurements are made at cruise altitude (between 9 and 12 km), CALIPSO scans the entire stratospheric aerosol column. The distribution of aerosol produced by the eruption of Kasatochi is clearly shown by the scattering ratio (the ratio of the measured scattering to the modelled molecular scattering), an optical equivalent to the mixing ratio (Fig. 2). The eruption injected ash and gases into two layers: one above 15 km that eventually spread over the entire NH, and one below 15 km in the NH LMS and extratropical UT. During the first few weeks after the eruption, the amount of aerosol increased due to the conversion of SO_2_ into sulfate particles^31^,^32^. The volcanic particles produced in the LMS had almost vanished by November 2008 through export to the troposphere, from where they were efficiently removed. The LMS volcanic aerosol concentrations increased again after subsidence of the upper cloud (Fig. 2), thus explaining the elevated S/O_3_ ratios observed by CARIBIC in December 2008 (see inset in Fig. 1).

**Figure 3:**
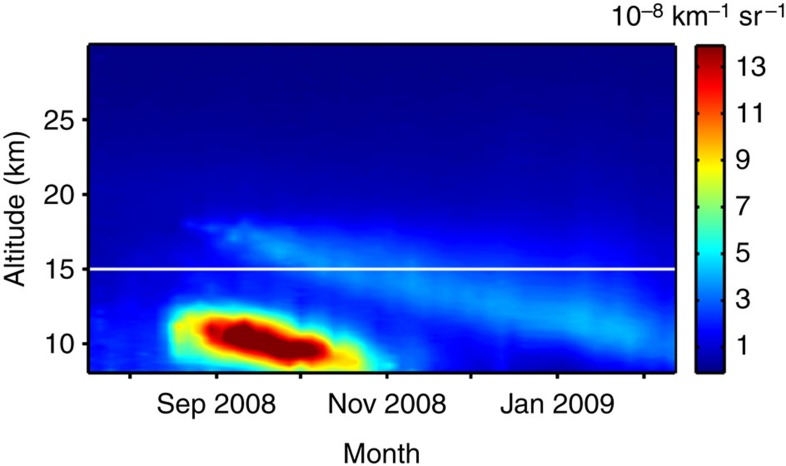
Downward transport of Kasatochi volcanic aerosol. Time evolution from July 2008 to Feb 2009 of aerosol scattering as a function of altitude, spatially averaged over 40° N–80° N. Tick marks relate to the first day of a month. Results are shown as total backscatter from CALIPSO minus molecular backscatter. The white line indicates 15-km altitude.

Most of the aerosol from the Kasatochi eruption is found in the lower volcanic cloud. This is clear from examining aerosol scattering averaged over the extratropics (Fig. 3), which is optically equivalent to the aerosol concentration. The lower volcanic cloud had a relatively short but very large effect on the aerosol concentrations in the LMS, lasting ∼2.5 months. The subsequent effect from the upper branch prolonged the volcanic influence on the LMS.

## Discussion

**Figure 4:**
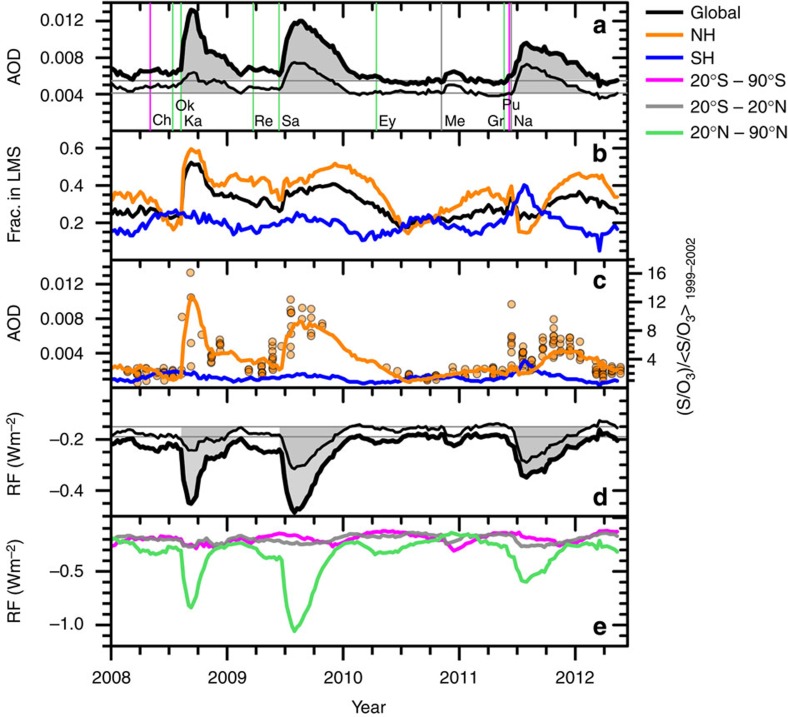
Volcanic influence on global and regional aerosol radiative parameters. (**a**) Global AOD at 532-nm wavelength calculated using the integrated CALIPSO aerosol scattering from 15 to 35-km altitude (thin line) and from the tropopause to 35 km (thick line). Major tick marks relate to Jan 1. The volcanic eruption dates are denoted by vertical lines, colour coded according to latitude. The eruptions are: Ch (Chaitén), Ok (Okmok), Ka (Kasatochi), Re (Redoubt), Sa (Sarychev), Ey (Eyjafjallajökull), Me (Merapi), Gr (Grimsvötn), Pu (Puyehue-Cordón Caulle) and Na (Nabro), see Table 1 for details. The grey horizontal lines indicate the estimated background AOD of the 2008 to mid-2012 time period, and the shading denotes the total integrated volcanic AOD from the Kasatochi, Sarychev and Nabro eruptions. (**b**) The fraction of total AOD from the LMS. (**c**) AOD in the LMS from CALIPSO (lines) and S/O_3_ in the NH from CARIBIC (circles). (**d**) As in **a**, but net radiative forcing (RF) calculated from AOD shown in **a**. (**e**) Stratospheric net radiative forcing in three regions equal in surface area.

In addition to the Kasatochi eruption, the Sarychev and Nabro eruptions (Table 1) clearly increased the global stratospheric AOD between 2008 and mid-2012, both above and below 15-km altitude (Fig. 4a). At least four other volcanic eruptions also had some influence on the stratospheric aerosol loading in this period. The contribution of the LMS to total stratospheric aerosol is evident from a comparison of the global AOD calculated for the 15–35-km altitude range (the range used in almost all previous studies) and the total AOD between the tropopause to 35 km (Fig. 4a). For August to November 2008, the lower limit for calculating the integrated AOD was set to 2 km below the tropopause to include the total effect from the Kasatochi eruption, since the aerosol produced partly resided in the UT (see above).

To estimate the impact from the three largest eruptions between 2008 and mid-2012, the background AOD and radiative forcing during this time period were set to the values in the relatively quiescent periods between the Kasatochi, Sarychev and Nabro eruptions. Time integration over the elevated AOD during the three periods (Fig. 4a) then provides the total influence (grey areas in Fig. 4a) for the duration of appreciable impact of an eruption. From the integrated stratospheric AODs without and with the LMS included for the Kasatochi, Sarychev and Nabro eruptions, we found that altitudes below 15 km accounted for large fractions of the integrated AOD from these eruptions, namely 68, 54 and 41%.

Next, we estimate the fraction of the total, global stratospheric AOD attributable to aerosol in the LMS (*f*_LMS_) over the entire 2008 to mid-2012 time period (Fig. 4b). The fraction is low (generally 20–30%) in periods between the three main eruptions. The *f*_LMS_ exceeds 50% 1 month after the Kasatochi eruption, and exceeds 40% after the Sarychev eruption. The Nabro eruption also yielded f_LMS_ values >30%. Our results for the years 2008–2011 show an average *f*_LMS_ of 30%. This result is at the lower limit of the previous estimate of 30–70% for the years 2000–2013 by Ridley *et al*^26^. There are a number possible explanations for this finding. Ridley *et al.*^26^ estimated stratospheric AOD based on data from the AERONET network of sun photometers. Because AERONET does not provide vertically resolved AOD data, this method has to rely on a model of the vertical distribution of the tropospheric AOD to estimate the stratospheric AOD. The time resolution of this approach is also limited, and the AOD impact of individual eruptions cannot be clearly resolved^26^.The vertically resolved measurements from CALIPSO and IAGOS-CARIBIC permit a clearer separation between stratospheric and tropospheric AOD, and provide a new and independent assessment of the radiative impact of aerosol in the LMS.

In the following, we investigate *f*_LMS_ of the two hemispheres. The *f*_LMS_ is generally higher in the NH, and clearly shows an identifiable influence from the major eruptions (Fig. 4b). Three of the 4 years analysed display deep minima of *f*_LMS_ in July to August, with the exception of 2009 (which is masked by the Sarychev eruption). This annual minimum coincides with the minimum in size of the NH LMS^16^ and the summer flushing of the LMS with tropical tropospheric air due to the weakened subtropical jet^17^.

The SH was influenced by the eruptions of Merapi and Puyehue-Cordón Caulle. The SH *f*_LMS_ shows an annual variation pattern that is briefly disrupted in 2011 by the Puyehue-Cordón Caulle eruption. As in the case of the NH, the SH *f*_LMS_ has minima during all 5 years. These are evident in January to March, coinciding with the SH LMS minimum in size. The small influence from volcanism in the SH makes further investigation of the stratosphere–troposphere exchange feasible, because a large fraction of the stratospheric aerosol is formed in the Junge layer from carbonyl sulfide (OCS). The fraction of the LMS air that originates in that part of the stratosphere varies over the year, from ∼60% in the winter spring to 20% in the summer autumn^33^,^34^. With roughly 40% of the stratospheric air mass found in the LMS^16^, we estimate that the LMS fraction of the aerosol-rich stratospheric air varies between 12 and 27% over the year. This estimate agrees well both in size and seasonal variation with our estimate of the observed *f*_LMS_ of the SH, indicating that during background conditions the LMS holds ∼20% of the stratospheric aerosol.

All of the volcanic eruptions studied here increased the relative importance of the LMS. We speculate that most volcanic eruptions affecting the stratosphere will probably induce such an increase. This is obvious for extratropical eruptions injecting SO_2_ directly to the LMS. Tropical eruptions can also be expected to increase *f*_LMS_. At background conditions, a large fraction of the stratospheric aerosol is formed from OCS in the deep branch of the Brewer–Dobson circulation at altitudes of 25–30 km, because intense ultraviolet radiation is required to oxidize OCS in a first step to form sulfuric acid aerosol. Formation of sulfate aerosol from volcanic SO_2_ takes place also at low stratospheric altitudes, which increases the aerosol transport in the shallow branch of the Brewer–Dobson circulation having a shorter residence time than the deep branch. A shorter residence time of the aerosol before entering the LMS increases the relative importance of the aerosol in the LMS.

Figure 4c shows the AOD of the SH and NH LMS obtained from CALIPSO LIDAR measurements, together with IAGOS-CARIBIC measurements of particulate sulfur. The IAGOS-CARIBIC measurements were taken in the NH, and were sampled in a strong concentration gradient arising from mixing of air across the extratropical tropopause. Because of this sharp gradient, the ratio to ozone (rather than the absolute concentration) is more representative of the volcanic additions to the LMS aerosol^29^. The IAGOS-CARIBIC measurements in proximity to volcanic eruptions exhibit large variability, which is subsequently reduced by atmospheric mixing. In contrast, the CALIPSO data are averaged hemispherically, thus smearing out small-scale spatial variability. This difference in spatial sampling is apparent after the Grimsvötn eruption, which causes a short but intense S/O_3_ peak in the aircraft measurements, but has only a small signature in the CALIPSO observations. The effluents of that eruption were injected in the UT and the tropopause region, thus explaining the rapid decline and the comparatively weak response in the CALIPSO measurements. In addition, most of the IAGOS-CARIBIC observations were made at latitudes >55° N, outside of the latitude limit for CALIPSO night-time measurements (see Fig. 2). Aside from this difference, the CALIPSO and IAGOS-CARIBIC measurements—which were made with completely different instruments—show similar temporal variability.

**Figure 5:**
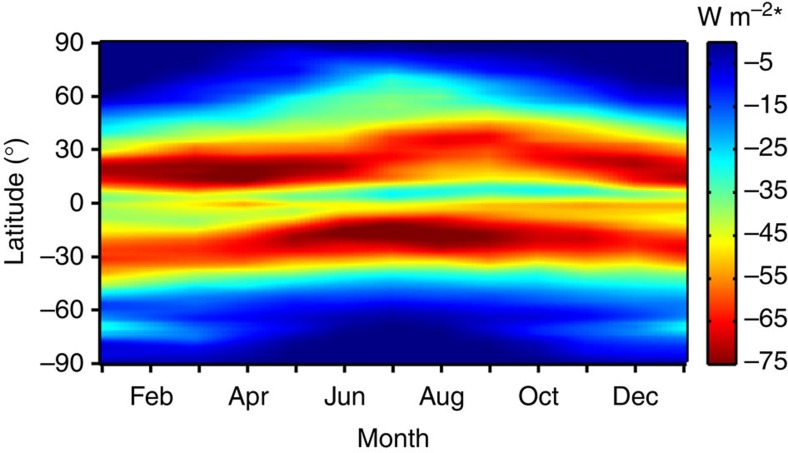
Radiative forcing sensitivity to stratospheric AOD. Geographical and seasonal dependences of the average net radiative forcing for AOD=1 at wavelength 532 nm. Tick marks relate to the first day of a month. Data are averaged over the period 2008–2011, and normalized by relative latitude area (*). The structures result from variability in daylength, upscatter fraction, surface albedo, cloudiness and transmission down to the aerosol layer.

In the following, we investigate the contribution of volcanic aerosol in the LMS region to recent climate change. We estimate this contribution by calculating radiative forcing using AOD estimates integrated over both 15–35 km and the tropopause −35-km ranges (Fig. 4d). Our calculations are a function of both season and latitude (Fig. 5). As in the case of the AOD results described earlier, the radiative forcing is integrated for the Kasatochi, Sarychev and Nabro eruptions. We find that 56, 44 and 23% of the total radiative forcing from these eruptions originated from below 15 km.

The relationship between AOD and radiative forcing is affected by a variety of factors, including the latitudinal and seasonal variation in cloud cover, surface albedo and solar zenith angle. The extratropical location of the LMS introduces a dependence of radiative forcing on the seasonal variation in the number of sunlight hours. For Kasatochi, a clear elevation of the LMS AOD (defined here as the time period when the LMS AOD exceeded 50% of its peak value for a specific eruption) lasted from mid-August to the end of October. The influence from Sarychev extended from mid-2009 until the end of that year. This implies that both eruptions had their main impacts approximately centred on the time of the autumn equinox. The dominant part of the aerosol from the tropical Nabro eruption was advected north; Nabro's main impact on the LMS AOD occurred from the end of August to mid-February. Since this time of the year is characterized by few daylight hours, the radiative impact of the Nabro eruption in the LMS region was markedly reduced.

The eruptions studied here have clear geographical signatures (Fig. 4e). The NH extratropics were most affected by the three large eruptions described above, even in case of the near-equatorial Nabro eruption. A complete description of the volcanic aerosol loading from the total stratosphere, including the LMS region, will improve our understanding of the global- and regional-scale climatic effects of recent volcanic activity^5^. Such information will be useful for studies seeking to quantify the role of volcanism in the present ‘slow-down' in global-mean surface warming^2^.

The IPCC best estimate of volcanically induced radiative forcing (from ref. 35) is based only on stratospheric measurements above 380 K in potential temperature^12^, resulting in an aerosol radiative forcing of −0.11 (−0.15 to −0.08) W m^−2^ during the period 2008–2011. Our results indicate that inclusion of the LMS increases the global stratospheric AOD by 45% and global radiative forcing by >30% during this 4-year period. This substantially increases the estimated radiative forcing and surface cooling. These findings provide considerable motivation for repeating CMIP5 simulations of historical climate change with improved estimates of AOD for the total stratosphere.

## Methods

### IAGOS-CARIBIC sampling and analysis

We used data from the IAGOS-CARIBIC observatory ( www.caribic-atmospheric.com) which is based on a 1.5 ton measurement container transported onboard a long-range passenger aircraft. Measurements are typically made during four consecutive flights per month^28^. Aerosol sampling in IAGOS-CARIBIC is based on impaction of particles of 0.08–2.0-μm diameter onto 0.2-μm-thick polyimide films^36^. The time resolution is 100 min (150 min before 2005), which corresponds to a spatial resolution of ∼1,500 km. Collected particles were analysed for elemental composition by two accelerator-based methods: Particle Induced X-ray Emission and Particle Elastic Scattering Analysis^37^ at the Lund ion beam accelerator facility. The accuracy is estimated to be 10% (ref. 38). We also use O_3_ mixing ratios obtained from IAGOS-CARIBIC, which have an accuracy of 0.3–1% (ref. 39). Particle size distributions were measured in 16 size channels in the diameter range of 0.13–0.9 μm with an optical particle counter^38^. The dynamical tropopause was used for classification of samples, where those taken in air masses with average potential vorticity (PV) >2 PVU (1 PVU=10^−6^ K m^2^ kg^−1^ s^−1^) were classified as stratospheric. PV was obtained from ECMWF (European Centre for Medium-Range Weather Forecasts (ECMWF, http://www.ecmwf.int/)) reanalysis data at a 1 × 1 degree horizontal resolution and 91 vertical hybrid sigma-pressure levels.

### CALIPSO data processing

The CALIPSO satellite performs ∼15 orbits per day, with a 16 day repeat cycle, and covers the globe between 82° S and 82° N (ref. 27). We use lidar data from the CALIPSO Level 1 night-time output of the 532-nm parallel and perpendicular polarized channels. Data were processed based on the method developed by Vernier, *et al.*^40^, including a shift of aerosol-free reference altitude from 30–34 km to 36–39 km. Each satellite swath was averaged horizontally to 1° latitudinal resolution and 180-m vertical resolution. Cloud pixels were identified and removed using a 5% threshold on the depolarization ratio to create a cloud mask^40^. The cloud mask was expanded upwards by 360 m to reduce the probability of missing faint upper edges of the clouds, and downwards towards the surface to remove attenuated signals from below the cloud. The final products used in this study are scattering ratios (measured total backscatter divided by calculated molecular backscatter) and aerosol scattering (measured total backscatter minus calculated molecular backscatter). The molecular scattering was modelled based on air and ozone molecule number concentrations^41^,^42^, using ozone number density and pressure from the Global Modelling and Assimilation Office (GMAO, http://gmao.gsfc.nasa.gov/) and temperature from the ECMWF. The aerosol scattering and scattering ratios were further averaged longitudinally over swaths from several days to achieve a time resolution of 8 days or 1 month.

### Aerosol optical depth

The conversion of aerosol scattering to AOD is dependent on particle size distribution. IAGOS-CARIBIC size distributions are available from mid-2010. The particle number concentrations increased appreciably after the Nabro eruption, and the size distributions shift slightly towards larger sizes compared with periods of low volcanic influence. However, the latter differences are small and all the size distributions are similar to that of the stratospheric background aerosol in 1999 (ref. 43). Therefore, the stratospheric background size distribution^43^ was used for the entire period with the conversion factor 50 between aerosol scattering and particle extinction^44^.

AOD is obtained by integration over the atmospheric depth. Because the tropopause altitude varies in time and space, zonal averaging was performed over (appropriately weighted) latitudinally varying altitude ranges. Wintertime data at latitudes ranging from 60° S to 90° S were removed because of frequent occurrence of polar stratospheric clouds that could not be effectively screened by the cloud mask. Polar stratospheric clouds in the NH occurred much less frequently and could be identified and removed manually. Further restrictions on data availability at high latitudes arise from the fact that CALIPSO night-time data do not extend to the poles in the summer season (the maximum latitudinal extent ranges from 55° to 80° over the year). The scattering of these regions were estimated based on extrapolation of neighbouring data. Due to the relatively small surface area contribution from high latitudes (∼7% from 60° to 90°S/N) uncertainties of actual aerosol concentrations in these regions are expected to have a relatively small effect on the calculated global AOD.

### Radiative forcing

A simple box model^45^ was used to calculate short-wave instantaneous radiative forcing (Δ*F*) for AODs derived from CALIPSO data:


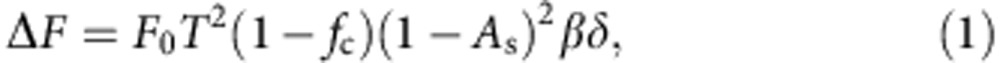


where *F*_0_=1,361 Wm^−2^ is the solar constant, *A*_s_ is the surface albedo, *f*_c_ the cloud fraction, *β* the upscatter fraction and *δ* the AOD. For the calculations of two-way transmission (*T*^2^) of incident light above an aerosol layer, the stratospheric aerosol was approximated as thin layers at 12.5 and 17.5-km altitude to represent average aerosol altitudes in the LMS and between 15 and 20 km. The net radiative forcing (including the long-wave component) is estimated to be 70% of the short-wave forcing^46^. The monthly mean surface albedo and cloud fraction were obtained from the ECMWF ERA-interim meteorological reanalysis project^47^. The two-way transmission was calculated at 532 nm as a function of solar zenith angle. Upscatter fractions for sulfuric acid particles as a function of particle size and solar zenith angle were deduced from Nemesure *et al*.^48^ Extinction coefficients of solar radiation on sulfuric acid aerosol were calculated, using the size distribution of background aerosol in 1999 (ref. 43). The extinction coefficients were used to integrate over the upscatter fraction for aerosol particles with radii in the range 0.029–0.679 μm.

The resulting radiative forcing is thus sensitive to variation in latitude and season through the dependence on daylight hours, solar zenith angle and the variation in cloud cover and surface albedo (Fig. 5), whereas the effect of the zenith angle on solar insolation and AOD cancel each other out^48^. The sensitivity of the radiative forcing to the AOD is obtained from the distribution in Fig. 5, where the global average radiative forcing for AOD=1 is −23 W m^−2^, consistent with data from literature^49^.

## Additional information

**How to cite this article:** Andersson, S. M. *et al.* Significant radiative impact of volcanic aerosol in the lowermost stratosphere. *Nat. Commun.* 6:7692 doi: 10.1038/ncomms8692 (2015).

## Figures and Tables

**Table 1:** Volcanic eruptions in the 21th century that affect (or have the potential to affect) the aerosol loading of the stratosphere.

**Volcano**		**Date**	**Lat.**	**Long.**	VEI*	**SO**_**2**_ **(Tg)**
Ulawun	Ul	29 Sep 2000	5° S	151° E	4	†
Sheveluch	Sh	22 May 2001	57° N	161° E	4	†
Ruang	Ru	25 Sep 2002	2° N	125° E	4	0.03 (ref. 50)
Reventador	Ra	3 Nov 2002	0° S	78° W	4	0.07 (ref. 50
Anatahan	At	10 May 2003	16° N	146° E	3	0.03 (ref. 50)
Manam	Ma	27 Jan 2005	4° S	145° E	4	0.09 (ref. 50)
Sierra Negra	Si	22 Oct 2005	1° S	91° W	3	†
Soufrière Hills	So	20 May 2006	17° N	62° W	3	0.2 (ref. 51)
Rabaul	Rb	7 Oct 2006	4° S	152° E	4	0.2 (ref. 50)
Jebel at Tair	Je	30 Sep 2007	16° N	42° E	3	0.08 (ref. 52)
Chaitén	Ch	2 May 2008	43° S	73° W	4	0.01 (ref. 53)
Okmok	Ok	12 Jul 2008	53° N	168° W	4	0.1 (ref. 52)
Kasatochi	Ka	7 Aug 2008	52° N	176° W	4	1.7 (ref. 52)
Redoubt	Re	23 Mar 2009	60° N	153° W	3	0.01 (ref. 54)
Sarychev	Sa	12 Jun 2009	48° N	153° E	4	1.2 (ref. 55)
Eyjafjallajökull	Ey	14 Apr 2010	64° N	20° W	4	†
Merapi	Me	5 Nov 2010	8° S	110° E	4	0.4 (ref. 56)
Grimsvötn	Gr	21 May 2011	64° N	17° W	4	0.4 (ref. 57)
Puyehue-Cordón Caulle	Pu	6 Jun 2011	41° S	72° W	5	0.3 (ref. 57)
Nabro	Na	12 Jun 2011	13° N	42° E	4	1.5 (ref. 57)

Lat., latitude; Long., longitude.

^*^VEI=Volcanic Explosivity Index (from Global Volcanism Program (http://www.volcano.si.edu/)).

^†^Not available.
